# Self-Assembling Nanoparticles Containing Dexamethasone as a Novel Therapy in Allergic Airways Inflammation

**DOI:** 10.1371/journal.pone.0077730

**Published:** 2013-10-25

**Authors:** Nicholas J. Kenyon, Jennifer M. Bratt, Joyce Lee, Juntao Luo, Lisa M. Franzi, Amir A. Zeki, Kit S. Lam

**Affiliations:** 1 Department of Internal Medicine, University of California Davis, Davis, California, United States of America; 2 Department of Biochemistry and Molecular Medicine, University of California Davis, Davis, California, United States of America; University ofTennessee Health Science Center, United States of America

## Abstract

Nanocarriers can deliver a wide variety of drugs, target them to sites of interest, and protect them from degradation and inactivation by the body. They have the capacity to improve drug action and decrease undesirable systemic effects. We have previously developed a well-defined non-toxic PEG-dendritic block telodendrimer for successful delivery of chemotherapeutics agents and, in these studies, we apply this technology for therapeutic development in asthma. In these proof-of-concept experiments, we hypothesized that dexamethasone contained in self-assembling nanoparticles (Dex-NP) and delivered systemically would target the lung and decrease allergic lung inflammation and airways hyper-responsiveness to a greater degree than equivalent doses of dexamethasone (Dex) alone. We found that ovalbumin (Ova)-exposed mice treated with Dex-NP had significantly fewer total cells (2.78±0.44×10^5^ (n = 18) vs. 5.98±1.3×10^5^ (n = 13), P<0.05) and eosinophils (1.09±0.28×10^5^ (n = 18) vs. 2.94±0.6×10^5^ (n = 12), p<0.05) in the lung lavage than Ova-exposed mice alone. Also, lower levels of the inflammatory cytokines IL-4 (3.43±1.2 (n = 11) vs. 8.56±2.1 (n = 8) pg/ml, p<0.05) and MCP-1 (13.1±3.6 (n = 8) vs. 28.8±8.7 (n = 10) pg/ml, p<0.05) were found in lungs of the Dex-NP compared to control, and they were not lower in the Dex alone group. In addition, respiratory system resistance was lower in the Dex-NP compared to the other Ova-exposed groups suggesting a better therapeutic effect on airways hyperresponsiveness. Taken together, these findings from early-stage drug development studies suggest that the encapsulation and protection of anti-inflammatory agents such as corticosteroids in nanoparticle formulations can improve efficacy. Further development of novel drugs in nanoparticles is warranted to explore potential treatments for chronic inflammatory diseases such as asthma.

## Introduction

Asthma is a progressive inflammatory airways disease that leads to structural airway changes and debilitating symptoms in many children and adults. An estimated twenty-six million people in the United States have asthma, and for adults, asthma accounts for two million emergency room visits per year. The annual cost for asthma is nearly $15 billion in the U.S., and eighty percent of the direct costs of asthma derive from the 5–10% of these patients who have severe or difficult-to-control asthma [1]–[4]. Many of these patients do not achieve asthma control with present therapies and novel therapeutic agents with improved drug delivery systems are needed.

We have focused on designing novel drug candidates to treat allergic lower airways inflammation that is indicative of asthma. In a prior study, we determined that by designing a specific T-shaped PEGylated structure and attaching it to a α4β1 integrin inhibitor, we could improve the anti-inflammatory properties of the inhibitor in the lungs of mice [5]. In the present study, we addressed the potential for using nanocarriers to improve drug delivery to the in this same mouse model. Nanocarriers can deliver various types of drugs, protect them from degradation and inactivation upon administration, and increase the fraction of drug delivered to a target organ. They can provide a mechanism to decrease the undesirable systemic effects of drugs. Many nanoparticles have been investigated for drug delivery, but only a few have been approved by the FDA [6]. Professor Lam and colleagues developed a well-defined nontoxic PEG-dendritic block telodendrimer composed of polyethylene glycol, cholic acid and lysine for successful delivery of therapeutics [6]. The nanocarrier that we chose to test has a greater loading capacity and superior stability (longer than six months) than micelles reported in the literature [7] The carrier allows for the delivery of slow-release hydrophobic drug formulations, such as dexamethasone, directly to the lung. In these proof-of-concept experiments, we hypothesized that dexamethasone contained in protective, self-assembling nanoparticles (Dex-NP) and delivered systemically would target the lung and decrease allergic lung inflammation and airways hyper-responsiveness to a greater degree than equivalent doses of Dex alone.

## Materials and Methods

### Synthesis of Nanoparticle

Diamino polyethylene glycol (Boc-NH-PEG-NH_2_, MW = 2000 Da) was purchased from Rapp Polymere (*Tübingen*, Germany). Fmoc-D-Asp(Otbu)-OH, Fmoc-D-Lys(Boc)-OH, and Fmoc-Lys(Fmoc)-OH were purchased from Anaspec, Inc. Hydrophobic NIRF dye DiD (1,10-dioctadecyl-3,3,30,30-tetramethylindodicarbocyanine perchlorate, D-307), 4′, 6-diamidino-2-phenylindole (DAPI) and LysoTracker® Green DND-2 were purchased from Invitrogen. Dexamethasone, cholic acid, MTT [3-(4,5-dimethyldiazol-2-yl)-2,5 diphenyl tetrazolium bromide], endocytosis inhibitors including chlorpromazine hydrochloride, amiloride hydrochloride hydrate, filipin III and all other chemicals were purchased from Sigma-Aldrich (St. Louis, MO).

**Figure 1:**
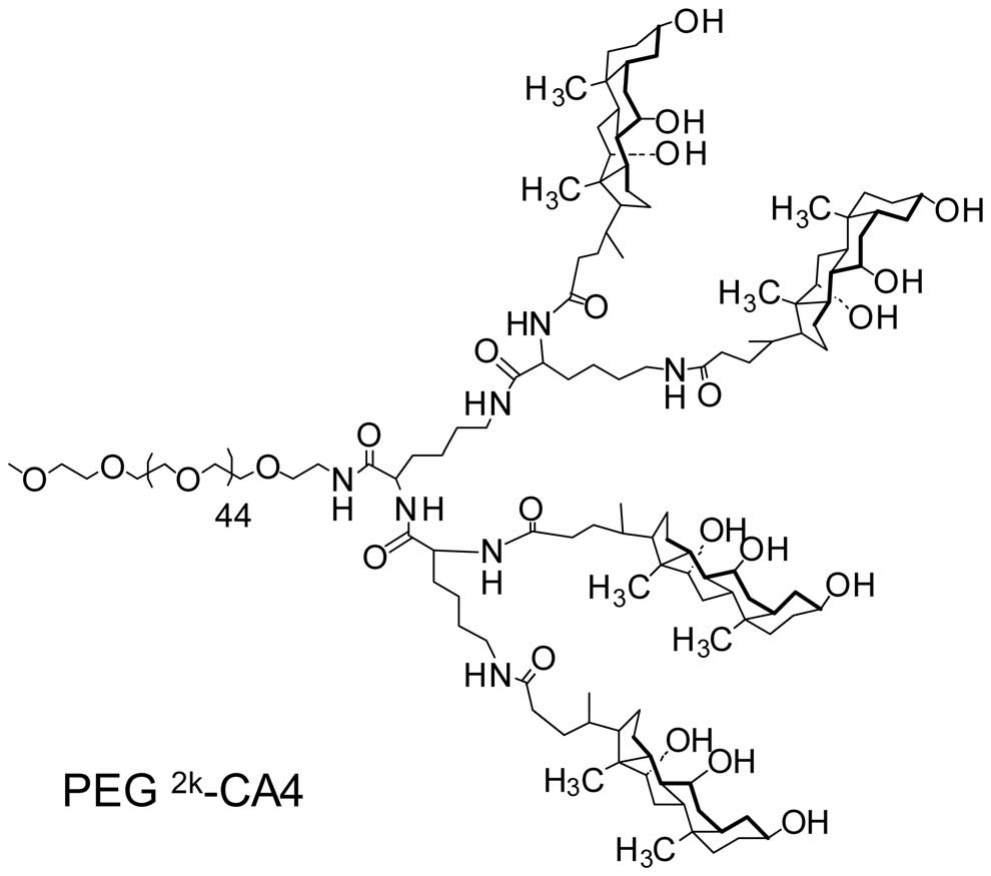
Diagram of PEG^2k^-CA4 telodendrimer. Boc-NH-PEG^2k^-CA4 telodendrimer was first synthesized using Boc-NH-PEG-NH_2_ (MW, 2000 Da), lysine and cholic acid as building blocks via solution phase condensation reactions. Fmoc peptide chemistry was used to couple Fmoc-Lys(Fmoc)-OH onto the unprotected amino group of PEG for three rounds to generate a third generation of dendritic polylysine.

Boc-NH-PEG^2k^-CA4 telodendrimer (**Fig. 1**) was first synthesized using Boc-NH-PEG-NH_2_ (MW, 2000 Da), lysine and cholic acid as building blocks via solution phase condensation reactions as described previously [6]. Briefly, Fmoc peptide chemistry was used to couple Fmoc-Lys(Fmoc)-OH onto the unprotected amino group of PEG for three rounds to generate a third generation of dendritic polylysine. Cholic acid NHS ester was coupled to the terminal end of dendritic polylysine, resulting in Boc-NH-PEG^2k^-CA_4_ telodendrimer. Then, the Boc group on the PEG chain of the telodendrimer was deprotected with 50% (v/v) trifluoroacetic acid (TFA) in dichloromethane (DCM), and different number (n = 0, 1, 3 and 6) of Fmoc-D-Asp(Otbu)-OH (d) or Fmoc-D-Lys(Boc)-OH (k) were subsequently conjugated to the distal end of PEG chain of PEG^5k^-CA_8_ telodendrimer by using Fmoc peptide chemistry. The primary amine at the N terminal of corresponding aspartic acids or lysines conjugated PEG^2k^-CA_4_ telodendrimer was acetylated by acetic anhydride. Finally, the Otbu groups of aspartic acids and Boc groups of lysines were removed to generate PEG^2k^-CA_4_ telodendrimer with different number of free carboxylic acids and primary amines, respectively. The telodendrimers were precipitated and washed three times with cold ether, dialyzed against water for 24 h and then lyophilized.

Characterization of the PEG^2k^-CA_4_ telodendrimer has been described previously [8]. Briefly, the morphology and particle size distribution of the telodendrimers loaded with the chemotherapeutic drug doxorubicin were characterized by transmission electron microscopy and direct light scatter techniques. The particle sizes of drug -loaded PEG^2k^-CA_4_ micelles were in the range of 10–20 nm in diameter and by electron microscopy, their shapes were spherical with an average diameter of around 15 nm, which was consistent with the results obtained from the light scatter particle sizer. Cumulative drug release profiles measured in dialysate showed that doxorubicin release from the PEG^2k^-CA_4_telodendrimer increased slowly from 30% to 60% between five to one hundred hours [8]. The characterization of this micelle has not been repeated with dexamethasone in these experiments.

### Animals

Mice (Balb/c, adult 8–12 wk. old males) were purchased from Charles River or Jackson Laboratories. Mice were certified as chronic respiratory disease free by the supplier, and are routinely screened for health status by serology and histology by our veterinary animal resources facility. There were no paramyxoviral, or other viral pathogens, present in these animals that might themselves provoke chronic airway inflammation. Animals were maintained in a HEPA-filtered laminar flow cage rack with a 12-hour light/dark cycle and allowed free access to food and water. Animals were housed and cared for by the veterinary staff of Animal Resource Services at University of California, Davis (UCD) in AALAC- accredited facilities, in plastic cages over autoclaved bedding in HEPA-filtered cage racks. Food (Purina Rodent Chow) and water are provided *ad libitum.* The mouse protocol and procedures were approved by the University of California, Davis Institutional Animal Care and Use Committee. In order to ensure animal comfort, mice were administered deeply sedating doses of medetomidine and tiletamine/zolpidem for non-terminal procedures, and lethal doses of pentobarbital at the time of euthanasia.

### Exposure of Mice to Ovalbumin Aerosol

Mice were divided into three air-exposed and three Ova-exposed treatment groups which varied in treatment by drug intervention only as described below. We developed an aerosol exposure apparatus suitable for exposure of mice to Ova aerosol after prior sensitization by i.p. injection of ovalbumin. Sensitization to Ova was developed by intraperitoneal injections on days 0 and 14 of chicken egg albumin (10 µg/0.1 mL ovalbumin, grade V, ≥98% pure, Sigma, St. Louis, MO) with alum as an adjuvant [9]. Exposure to Ova aerosol was performed using chambers and generators as described previously [10]. Aerosol exposures began on day 28. Mice were exposed for 30 minutes, three times per week for the duration of the experiment. Age-matched mice were exposed aerosols derived from 10 ml phosphate buffered saline, (pH 7.4, PBS), to 10 mL ovalbumin in PBS (10 mg/ml) or to filtered air. A side-stream nebulizer (Invacare Corporation, Elyria, OH), ProNeb compressor nebulizer (Pari, Richmond, VA), and Passport Compressor (Invacare, Sanford, FL) were used to generate the aerosols.

In our initial experiment, we determined that the size and shape of the PEGylated dendrimer did not affect dexamethasone-mediated response. Specifically, there was no difference in the anti-inflammatory effect of dexamethasone whether it was loaded in a PEG^2k^-CA_4_ or a PEG^5k^-CA_8_ telodendrimer. All subsequent experiments were performed with the PEG^2k^-CA_4_ telodendrimer. Thus, mice were administered either Dex-PEG^2k^-CA_4_ (Dex-NP; 5 mg/kg/day of Dex), the equivalent dose of Dex, or PEG^2k^-CA_4_ (NP) alone thirty minutes before exposure to either ovalbumin or filtered air for 7 days (3 ovalbumin exposures). The decision to use the intravenous route of administration with the PEGylated formulation was made after a consideration of the pharmacology of the newly synthesized antagonists, to address concerns that absorption of the larger PEGylated antagonists from a bolus dose i.p. might be impaired. Lung tissue and lavage samples were measured in all mice. To address the question of whether the PEG^2k^-CA_4_ affected lung cell counts caused hemolysis of red blood cells as earlier reported [6], we administered the nanoparticle compound in its empty formulation in animals prior to either filtered air or Ova.

### Lung Compliance and Resistance Measurements

We measured dynamic compliance and resistance of the respiratory system with a plethysmograph for restrained animals. (Buxco Inc., Troy, NY). Mice were deeply anesthetized and sedated with medetomidine, 0.5 mg/kg (Domitor, Orion Pharma, Finland), and tiletamine/zolpidem, 50 mg/kg (Telazol, Fort Dodge Laboratories, Fort Dodge, IA) and surgically cannulated. Mice were then inserted into the whole body plethysmograph and ventilated at 7–8 cc/kg with a mouse ventilator (MiniVent, Harvard Apparatus, Cambridge, MA) for the duration of the procedure. Compliance and resistance measurements were made at baseline and immediately following serial 3-minute nebulizations of saline and serial low doses of methacholine (0.5, 1.0 and 2.0 mg/ml).

### Measurement of Exhaled NO and Nitrate/Nitrite

A 5-minute sample of exhaled gases from the exhalation port of the ventilator was collected immediately after insertion of a mouse into the plethysmograph. Samples were collected into a specially constructed Tyvek bag from the cannulated mice. This 5-minute sample is adequate for the measurement of NO concentration in the expired air, using a Sievers Nitric Oxide analyzer (Sievers Inst., Boulder, CO) [11].

### Whole Lung Lavage

Mice were killed with an overdose of pentobarbital/dilantin one hour after cessation of the last aerosol exposure, their tracheas were cannulated, and their lungs were lavaged twice with 1 ml portions of phosphate-buffered saline, pH 7.4. The total volume of lavage fluid recovered averaged >80%. Collected lavage fluid was centrifuged in a bench top unit at 1200 rpm for 10 min and the resulting pellet was again suspended in 0.5 ml PBS. The final cell suspension was used to determine total lavaged cell number using a hemacytometer and calculate cell differentials using cytocentrifuge preparations. Aliquots (100 µl) of the cell suspension processed onto glass slides in a cytocentrifuge (1650 rpm for 15 minutes) Slides were then stained with Diff-Quick staining kit (International Reagent Corp, Kobe, Japan). Stained cells were classified as pulmonary alveolar macrophages, polymorphonuclear leukocytes (neutrophils), lymphocytes, or “other” based upon staining color and characteristic morphology. Blood smears were made to quantify the number of schistocytes and acanthocytes in the peripheral blood of animals. Results are presented as pooled data from the two independent experiments, which gave similar independent results.

### Lung Isolation and Fixation

After lung lavage, the lungs were fixed for histological evaluation at 30 cm pressure with 1% paraformaldehyde for at least 24 hours, then either processed for light microscopy in paraffin or stored in 70% ethanol for dissection and analysis of airways. Other lung preparations were dissected without fixation to prepare airways, which were frozen and stored at –80°C until used for extraction of RNA for PCR analysis.

### Tissue Staining

After 1 hour of in situ fixation, the lung removed from the body cavity, placed in 70% ethanol and prepared for paraffin embedding in a standard fashion. Lung sections of 5 µm thickness were made with special attention to cutting through the larger lobar bronchi in parallel. Sections were baked at 37°C overnight prior to staining. Lung sections were stained with Alcian Blue-Periodic Acid-Schiff (PAS) and counterstained with hematoxylin and eosin.

### Lactate Dehydrogenase (LDH) and Bilirubin Assay

Commercially available colorimetric kits were purchased to perform LDH activity and bilirubin measurements in plasma. These assays were used as indirect measures of intravascular hemolysis. Blood was drawn by cardiac puncture and centrifuged in K2 EDTA Plus blood collection tubes at 1200 rpm for 12 minutes. Plasma was aliquoted into 60 µl aliquots and stored, protected from light at 4°C until further use. LDH activity was determined in 2 µl plasma using the Lactate Dehydrogenase Activity Assay (Sigma-Aldrich, St. Louis, MO) as per manufacturer’s instructions using colorimetric detection at 450 nm. Bilirubin concentration in 50 µl plasma was determined using the Quantichrom Bilirubin Assay Kit (Bioassay Systems, Hayward, CA) as per manufacturer’s instructions. Assay measures the reaction of bilirubin with diazotized sulfanilic acid producing a red product that was measured at 540 nm. Caffeine benzoate was added to separate bilirubin from the unconjugated bilirubin protein complex to measure total plasma bilirubin.

### Cytokine and Chemokine Assay

The concentrations of selected Th1 and Th2 cytokines and chemokines from BALF supernatant were measured using commercially available multiplex assays (Millipore, St. Charles, MO, USA). For cytokine/chemokine sample measurements below the lower detection limit, results were assigned a value equal to the minimum detection limit for the specific assay to facilitate statistical analysis of the data.

### Statistical Analysis

Results are presented as mean values ± SEM. Means were compared by unpaired Student’s t-test or ANOVA (1-way or 2-way), with Tukey’s or Bonferroni correction for multiple comparisons applied where appropriate, using the Prism 5 software package (Graphpad, Inc., San Diego, CA). A p-value of 0.05 or less was taken to indicate statistical significance. Values that differed by more than two standard deviations from the mean were excluded from the statistical analysis. GraphPad Software (San Diego, CA) was used for data analysis. For parametric analysis of data, a t-test with appropriate correction for multiple comparisons and unequal standard deviations between groups was used. For non-parametric analysis, a Wilcoxin test was used.

## Results

**Figure 2:**
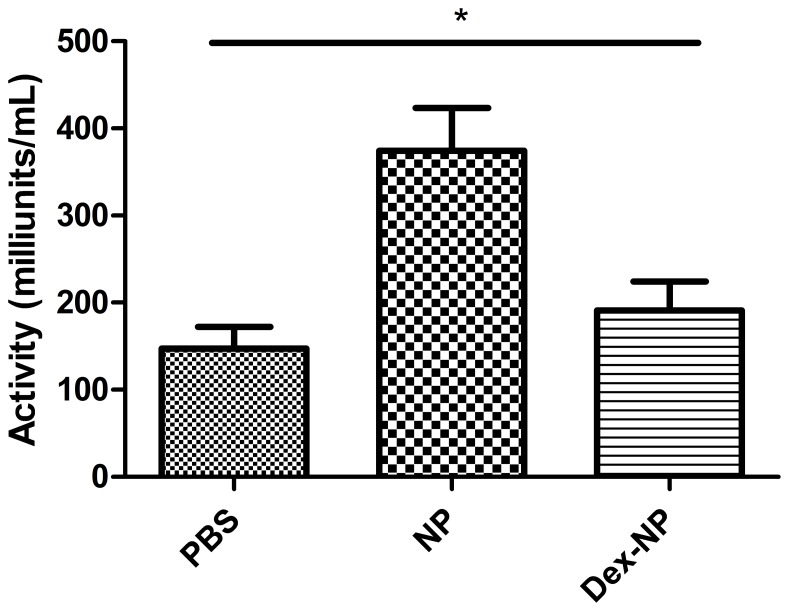
LDH activity was determined by a lactate dehydrogenase activity assay (Sigma-Aldrich, St. Louis, MO) as per manufacturer’s instructions using colorimetric detection at 450 nm. In this experiment, animals were not sensitized or exposed to ovalbumin. LDH activity was significantly higher in the plasma of the empty nanoparticle (NP) treated animals (374.2±98.9 milliunits/ml) compared to either the PBS control (147.3±49.65 milliunits/ml) or Dex-NP treated animal (191.1±66.2 milliunits/ml, p<0.01 by one-way ANOVA).

In a series of three separate experiments, Balb/c mice were exposed to inhaled ovalbumin or to filtered air three times over seven days and treated with either 5 mg/kg of Dex, 5 mg/kg of Dex loaded in -PEG^2k^-CA_4_ nanoparticles (Dex-NP), or empty nanoparticles (NP) intravenously thirty minutes before each of the three exposures. None of the mice had any gross observable toxicity related to the nanoparticle compound and the lung appears normal. There was not an increase in schistocytes on peripheral blood smears in the empty nanoparticle formulation treated animals, which had been a prior concern [12]. However, LDH activity was significantly higher in the plasma of the empty nanoparticle (NP) treated animals (374.2±98.9 milliunits/ml) compared to either the PBS control (147.3±49.65 milliunits/ml) or Dex-NP treated animal (191.1±66.2 milliunits/ml, p<0.01 by one-way ANOVA, **Fig. 2**). There was a trend to increased direct bilirubin in both NP treated groups (1.6±0.3 mg/dl (PBS) vs. 10.9±6.5 mg/dl (NP) vs. 7.0±6.4 (Dex-NP), but these differences were not statistically significant (n = 4/group, p = 0.09 by 1-way ANOVA). Furthermore, there was no difference between the PBS and NP groups by student’s t-test (1.6+/−0.3 vs. 10.9+/−6.5 respectively, p = 0.20). While there were no significant differences in plasma bilirubin content among the groups, we believe this assay was compromised by the turbidity of the plasma in all NP and Dex-NP treated animals which may be complicated the interpretation of the bilirubin readings difficult. Turbidity of the plasma was not an issue in the highly sensitive LDH assay which could be diluted substantially.

### Effects of Ovalbumin Exposure on Lung Inflammatory Response

**Figure 3:**
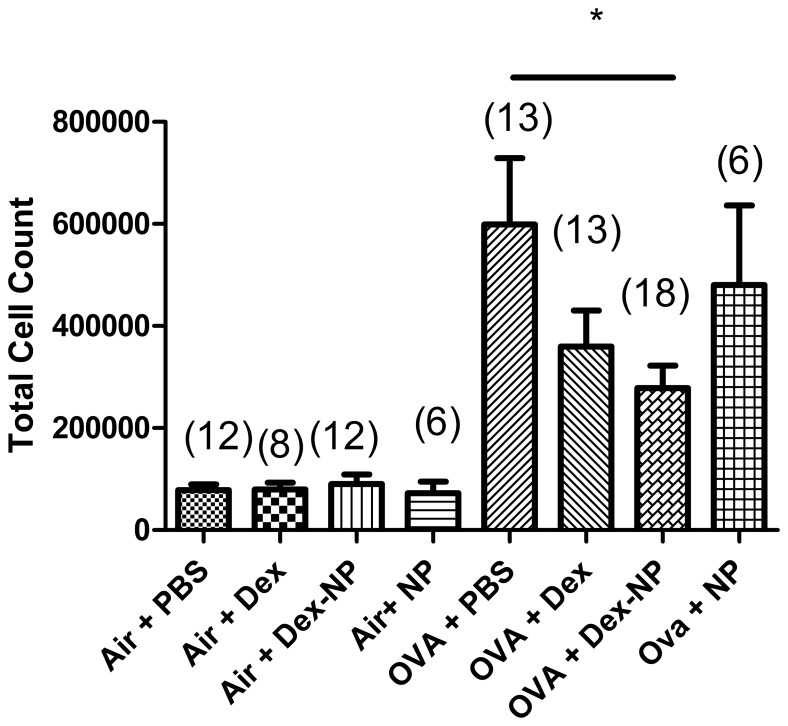
Total cells recovered by lung lavage from Balb/c mice exposed to filtered air or Ova aerosol for 1 week. Of the filtered air exposed mice, the mean cells present in their lavage was 8.21±0.8×10^4^ cells (pooled data from all groups, n = 41). There was a significant increase in the number of total lung lavage cells in each of the four ovalbumin exposed groups compared to the corresponding air-exposed animals (p<0.01 compared to all Ova groups). Ova-exposed mice treated with Dex-NP had significantly fewer total cells in the lung lavage than Ova-exposed mice (PBS-treated) alone (2.78±0.44×105 (n = 18) vs. 5.98±1.3×105 (n = 13) respectively, P = 0.013). Data are presented as mean values±SEM. *denotes p<0.05 and analyzed by Student’s T-test.

In all mice sensitized and exposed to ovalbumin, we observed a significant increase in the numbers of inflammatory cells recovered by lung lavage compared to mice exposed to filtered air alone, consistent with prior studies from our lab [13]–[15]. (**Fig. 3**). The number of lung lavage cells from the groups of mice exposed to filtered air from all time points evaluated was 8.21±0.8×10^4^ cells (pooled data from all groups, n = 41). The normal lung lavage from a healthy mouse contains more than 95% alveolar macrophages and our observations were consistent with this finding. There was a significant increase in the number of total lung lavage cells in each of the four ovalbumin exposed groups compared to the corresponding air-exposed animals. Ova-exposed mice treated with Dex-NP(Ova Dex-NP) had significantly fewer total cells in the lung lavage than Ova-exposed mice (Ova PBS) alone (2.78±0.44×105 (n = 18) vs. 5.98±1.3×105 (n = 13) respectively, P = 0.013). While the Dex alone treated group (Ova Dex) did demonstrate an expected trend towards decreased cell counts (3.59±10.7×10^5^ (n = 13)) compared to the Ova PBS control group, this was not statistically significant. Furthermore, Ova-exposed mice treated with empty NP treated animals (Ova NP) did not have fewer inflammatory cells in their lung lavage fluid than the Ova PBS controls.

**Figure 4:**
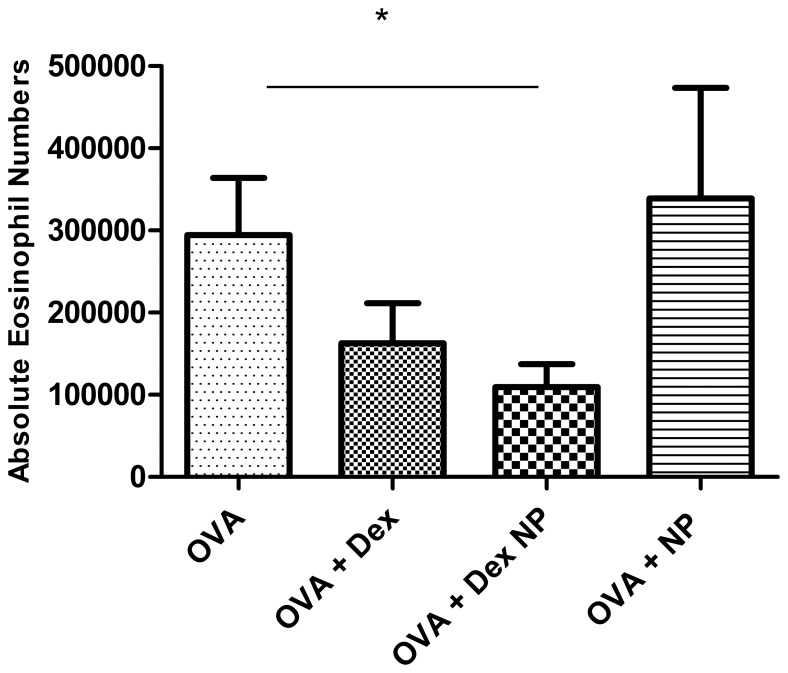
Total eosinophils recovered by lung lavage from Balb/c mice exposed to Ova aerosol for 1 week. Eosinophils comprised a significant proportion of the cells present in the lavage of mice exposed to Ova. Ova-exposed mice treated with Dex-NP had fewer eosinophils compared to PBS control mice (1.09±0.28×105 (n = 18) vs. 2.94±0.6×105 (n = 12) respectively, P = 0.016). Data are presented as mean values±SEM. *denotes p<0.05 by Student’s T-test.

Similarly, lung lavage eosinophil counts were significantly lower in the Ova Dex-NP animals. Ova Dex-NP mice versus Ova PBS control mice had 1.09±0.28×105 (n = 18) vs. 2.94±0.6×105 (n = 12) respectively, P = 0.016, **Fig. 4**). While the Ova Dex treated group did demonstrate an expected trend towards decreased eosinophil cell counts compared to the PBS-treated control group, again this was not statistically significant. There were no significant differences in the numbers of lymphocytes or neutrophils in the BALF among the groups. Collectively, these results suggest strongly that intravenous Dex-NP is more efficacious in preventing eosinophilic or allergic lung inflammation than the equivalent dose of Dex alone in Ova-exposed mice using this model.

### Respiratory System Resistance and Compliance

**Figure 5:**
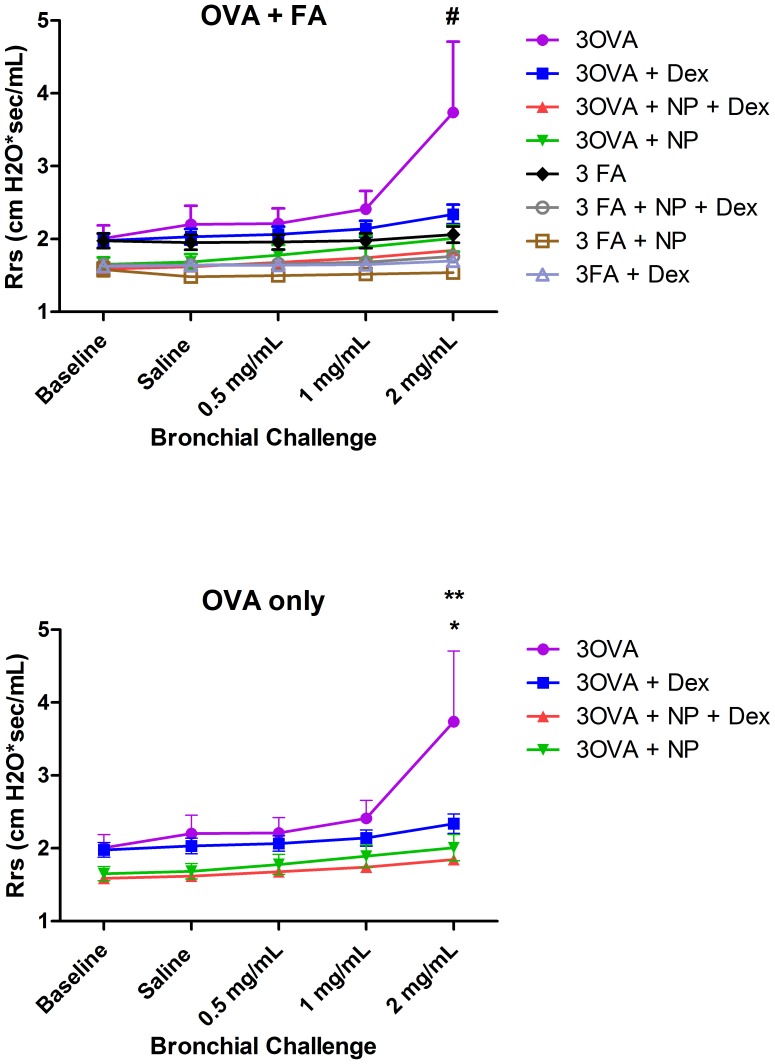
Total respiratory system resistance in Balb/mice exposed to either filtered air or 1 week of Ova (a) or Ova alone (b). Statistical comparisons were made to determine the effect of Dex and the nanoparticle status on resistance and compliance at baseline and after inhalation of saline (vehicle) and serial low doses of methacholine (0.5, 1.0 and 2.0 mg/mL). In the OVA group, treatment with either Dex or its nanoparticle drug vehicle (NP) independently attenuated Rrs and AHR (*,**p<0.0001) down to air control levels at the highest dose of methacholine. Rrs was significantly lower in the Dex-NP treated animals across all time points (F = 2.57, P<0.05), which supports the notion that it was more efficacious in preventing the both the inflammatory and AHR changes typically seen in this model. Data from all eight treatment groups were analyzed simultaneously using 2-way ANOVA with Bonferroni correction for multiple comparisons.

**Figure 6:**
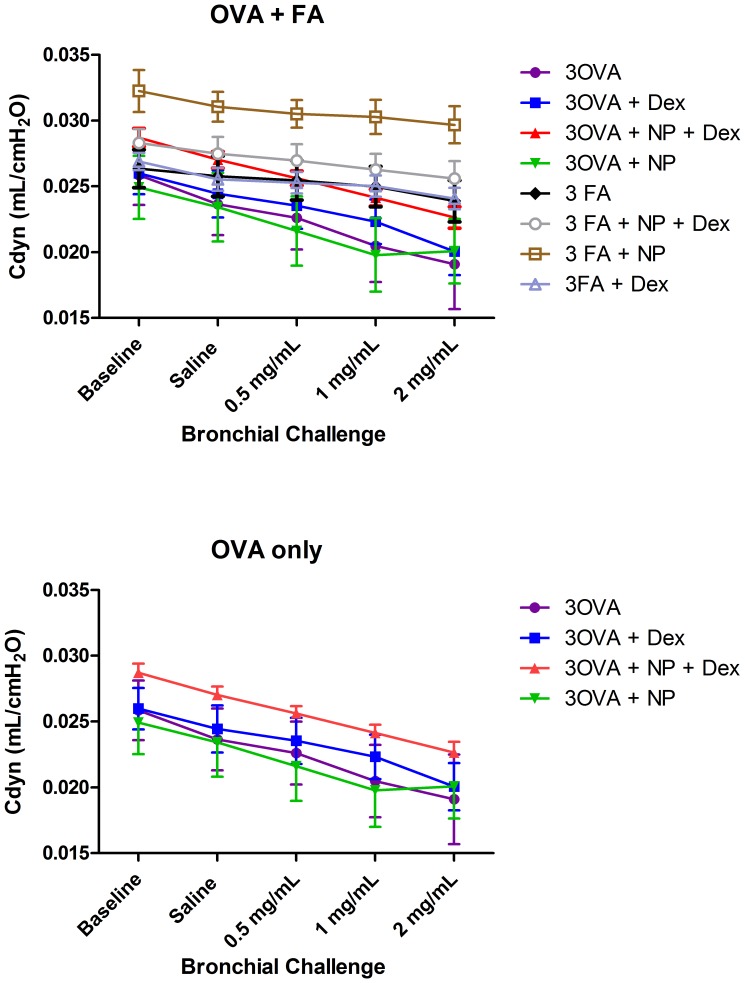
Total lung compliance in mice exposed to either filtered air or 2 weeks of OVA (a) or Ova alone (b). Lung compliance was measured at baseline and following serial doses (0, 0.5, 1.0 and 2.0 mg/ml) of nebulized methacholine (MCh). There was no difference in the slope of the MCh response among any of the groups of mice. Data from all eight treatment groups were analyzed simultaneously using 2-way ANOVA with Bonferroni correction for multiple comparisons.

Since airway hyper-responsiveness (AHR) is a fundamental feature of asthma, we compared the total lung resistance and dynamic compliance at baseline and after inhalation of methacholine in mice after the final ovalbumin exposure. Statistical comparisons were made to determine the effect of Dex and the nanoparticle status on resistance and compliance at baseline, after inhalation of aerosolized saline (vehicle), and serial low doses of aerosolized methacholine (0.5, 1.0 and 2.0 mg/mL). Data from all eight treatment groups were analyzed simultaneously using 2-way ANOVA with Bonferroni correction for multiple comparisons. There was evidence of a significant interaction among the groups (p<0.0001) when analyzed collectively. Inhalational challenge with OVA increased Rrs and AHR above air controls in response to methacholine (MCh) at 2 mg/mL indicating an adequate airway response to Ova in our model #p<0.0001). In the OVA group, treatment with either Ova Dex or its nanoparticle drug vehicle (Ova NP) independently attenuated Rrs and AHR (Fig. 5*,**p<0.0001) down to air control levels at the highest dose of methacholine. (**Fig. 6**). Rrs was significantly lower in the Ova Dex-NP compared to the Ova Dex treated animals when analyzed by linear regression (F = 2.57, P<0.05), which supports the notion that it was more efficacious in preventing the both the inflammatory and AHR changes typically seen in this model.

While AHR is the hallmark of the physiological changes in this mouse model, we also examined the effect of ovalbumin and the treatment on total dynamic compliance (Cdyn) of the respiratory system.

There were no differences in Cdyn among groups of mice exposed to either air or Ova. This finding suggests that the lung inflammation and injury were not the primary determinants of the physiological changes seen with the resistance measurements.

### Lung lavage Cytokines

**Figure 7:**
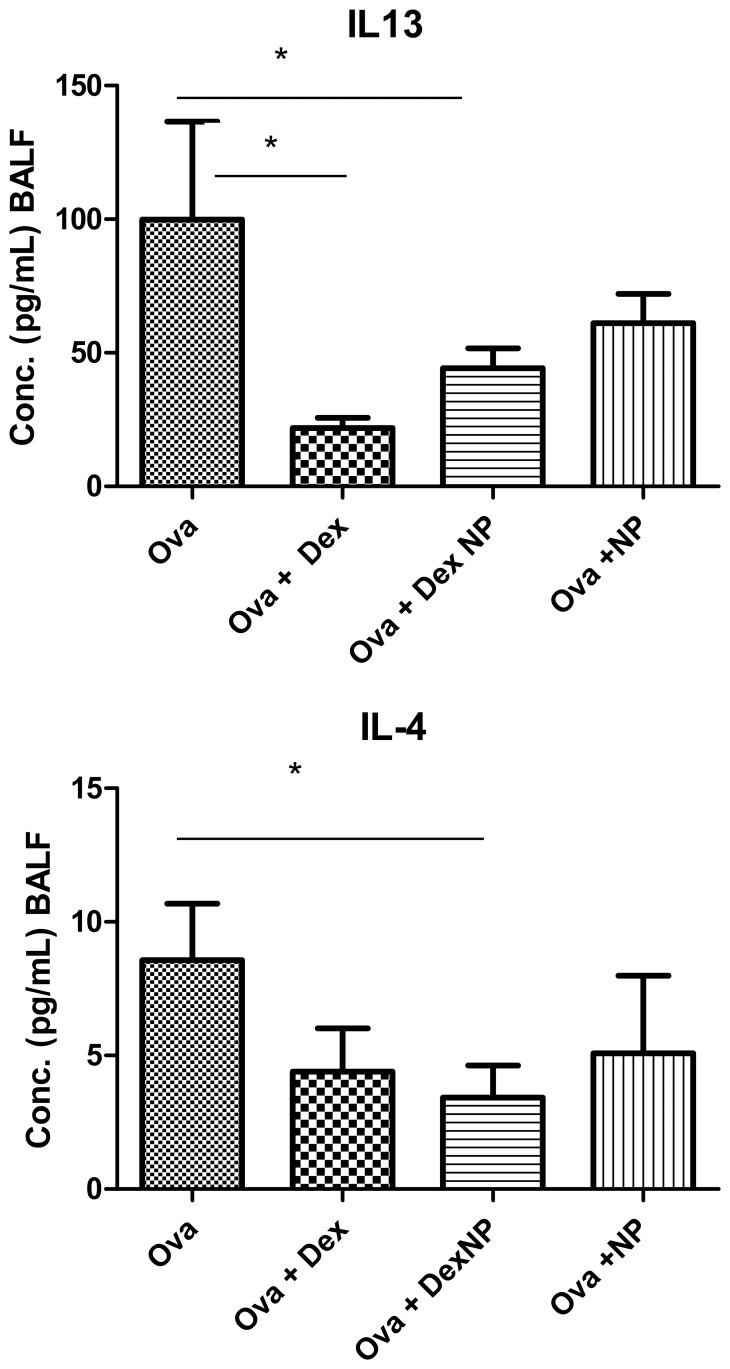
Lung lavage concentrations of IL-4 and IL-13 in mice exposed to air and Ova (a and b). In OVA-challenged animals, Dex-NP significantly decreased the lung lavage content of IL-4 compared to PBS control (3.43±1.2 (n = 11) vs. 8.56±2.1 (n = 8) pg/ml, p<0.05), while Dex alone did not. IL-13 levels in lung lavage fluid were significantly decreased in only the Dex treated animals compared to the Ova control animals. Data are presented as mean values±SEM. *denotes p<0.05 by Student’s T-test.

We examined several Th1 and Th2 cytokines in lung lavage fluid to determine whether dexamethasone containing nanoparticles affected the production of mediators important in allergic asthma. In OVA-challenged animals, Dex-NP(Ova Dex-NP) significantly decreased the lung lavage content of IL-4 compared to Ova PBS controls (3.43±1.2 (n = 11) vs. 8.56±2.1 (n = 8) pg/ml, p<0.05) (**Fig. 7**), while Dex alone (Ova Dex) did not. IL-13 levels in lung lavage fluid were significantly decreased in only the Dex treated animals compared to the Ova control animals. Neither BALF IL-4 levels (3.4±1.2 vs. 5.1±2.9 pg/ml, p = 0.6) nor BALF IL-13 levels (44.3±7.3 (n = 10) vs. 61.1±10.9 (n = 5) pg/ml, p = 0.2) were lower in the Ova Dex-NP animals compared to the Ova-NP animals. Furthermore, BALF IL-4 levels were not lower in the Ova-NP animals compared to the Ova animals (5.1±6.5 (n = 5) vs. 8.56±2.1 (n = 8) pg/ml, p = 0.12), while the Ova Dex-NP animals did have lower IL-4 levels than the Ova animals. Similarly, BALF IL-13 levels were not lower in the Ova+NP animals compared to the Ova animals (61.1±10.9 (n = 5) vs. 99.9±36.6 (n = 8) pg/ml, p = 0.7), while the Ova Dex-NP animals did have lower IL-13 levels than the Ova animals. We believe that the primary reason that there were no differences found in these two inflammatory cytokines between the Ova Dex-NP and Ova-NP was the few animals included in the Ova-NP group. Given the variability in these assay read-outs, many more animals would need to be included to determine the presence or lack thereof of an anti-inflammatory effect of empty nanoparticles.

Furthermore, MCP-1 was significantly lower in the Ova Dex-NP treated animals compared to the Ova PBS control-treated animals (13.1±3.6 (n = 8) vs. 28.8±8.7 (n = 10) pg/ml, p<0.05). Ova Dex treated animals were not significantly different from the Ova PBS control group. For IP-10, both the Ova Dex (118±18.3 pg/ml) and the Ova Dex-NP (145.8±25.8 pg/ml) had lower concentrations in lung lavage fluid than the Ova control animals (292.2±54.8 pg/ml, p<0.05 for both). Neither Dex nor Dex-NP had any significant effect on BALF concentrations of eotaxin, IL-5, IL-6, IL-1a, IL-9, IL-10, IL-12, IL-17, or IFN-g, TNF, GM-CSF, MIP-1α, KC, IP-10, RANTES.

### Exhaled Nitric Oxide

There was a significant increase in FeNO (14.61±1.4 ppb) at 2 weeks only in the filtered air exposed mice treated with Dex-NP (FA Dex-NP) compared to the other groups (1-way ANOVA, p<0.05, data not shown). There were no other significant differences. We conclude exhaled nitric oxide did not correlate with the level of eosinophilic lung inflammation, which differs from what has been described in humans [16].

### Goblet Cell Hyperplasia

**Figure 8:**
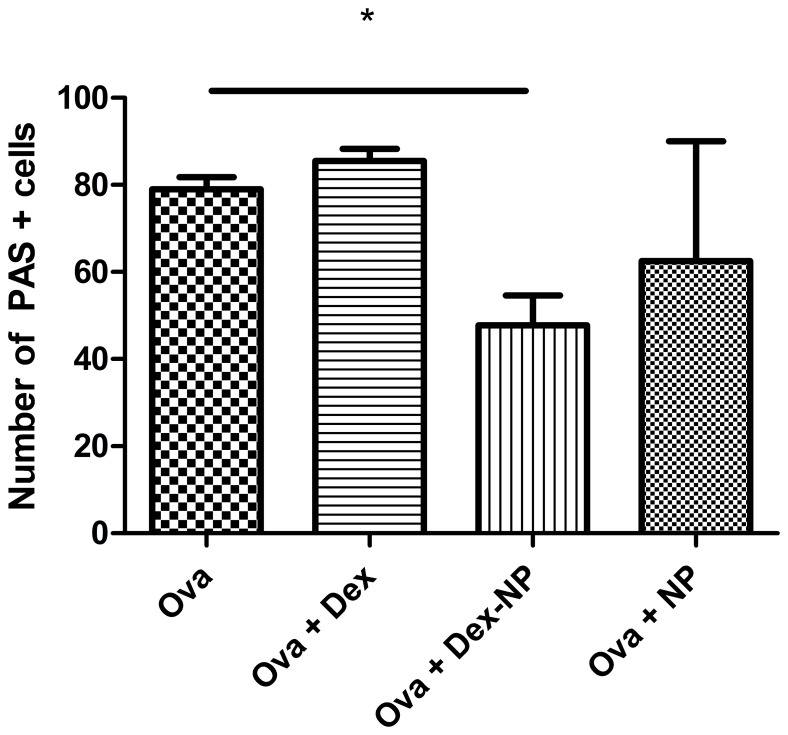
Number of PAS positive cells present in the airway epithelium among groups of mice exposed to Ova. PAS staining of 5 µm-thick, left lobe lung sections from select mice in each experimental group and images and counting of cells was done at 400×magnification. Filtered air exposed mice displayed an average of <1% PAS positive cells in the airway epithelium. The total number of PAS stained cells were quantified in the generation airway immediately branching from the lobar bronchus. PAS cells were counted per 100 basal airway epithelial cell nuclei. The number of positive PAS stained cells was significantly lower in the Dex-NP group than either the Dex (47.8±6.8 (n = 4) vs. 85.5±2.7 (n = 4) respectively, p<0.05, Fig. 6) or the control group (47.8±6.8 (n = 4) vs. 79.0±2.8 (n = 4), p<0.05) among the Ova exposed groups. Data are presented as mean values±SEM. *denotes p<0.05 by Student’s T-test.

**Figure 9:**
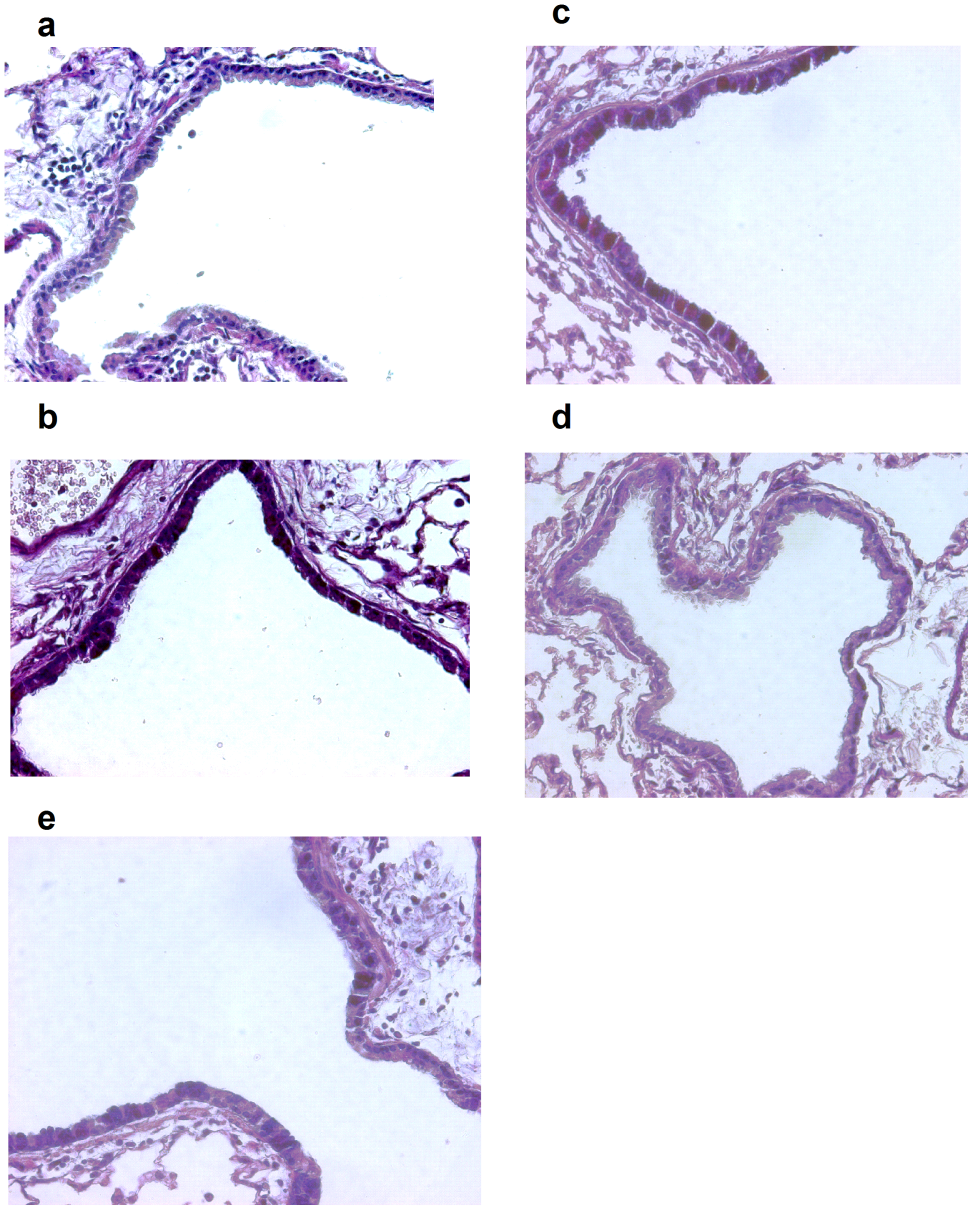
PAS staining for goblet cells from representative sections of lobar bronchi or daughter generation airway in mice from a) air-exposed, b) Ova-exposed (Ova), c) Ova-exposed Dex-treated (Dex), d) Ova-exposed Dex-nanoparticle (Dex NP), and e) Ova-exposed nanoparticle (NP) treated mice. Lung sections were stained with Alcian Blue-Periodic Acid-Schiff (PAS) and counterstained with hematoxylin and eosin and photographed at 400×magnification. PAS positive cells in airways were fewer in Dex NP treated animals.

To assess the effect of the nanoparticles containing dexamethasone on aspects of lung remodeling, we quantified goblet cell numbers by counting PAS positive stained cells. The total number of PAS stained cells were quantified in the generation airway immediately branching from the lobar bronchus. PAS cells were counted per 100 basal airway epithelial cell nuclei. The number of positive PAS stained cells was significantly lower in the Ova Dex-NP group than either the Ova Dex (47.8±6.8 (n = 4) vs. 85.5±2.7 (n = 4) respectively, p<0.05**,** **Fig. 8** **,** **Fig. 9a–d**) or the Ova PBS control group (47.8±6.8 (n = 4) vs. 79.0±2.8 (n = 4), p<0.05). Only two of the Dex-NP animals had sections fixed for staining and we cannot determine statistically whether there are fewer PAS positive cells in the airway epithelium compared to the Ova+Dex-NP animals. This reduction in goblet cell metaplasia in the Ova Dex-NP animals is consistent with the reduced inflammatory cell burden seen in these animals. Particularly, the lower IL-4 levels in the Ova Dex-NP may be responsible for the observed effect seen in the mucous cell amounts. While mucus extruded into the airway lumen was not quantified, it grossly appeared less in the Ova Dex-NP group by histology.

## Discussion

One hallmark of asthma is chronic allergic airway inflammation that is dominated by the effector leukocytes eosinophils, mast cells, and Th2 lymphocytes. Corticosteroids, particularly inhaled corticosteroids, are the standard of care for treating asthma and they are the basis of all treatment guidelines. Not surprisingly, in the Ova exposure mouse model, pretreatment with corticosteroids like dexamethasone leads to fewer lung lavage eosinophils than in mice treated with placebo. [17], [18] In the proof-of-concept experiments described here, we opted to use the corticosteroid dexamethasone. To address the issue of bioavailability of a hydrophobic corticosteroid like dexamethasone, we hypothesized that a pegylated, protected, and self-assembling nanoparticle would be more efficacious in preserving the function of these particles. The rationale was that a pegylated compound would remain in the blood circulation longer than the non-PEGylated formulations and that the nanoparticle would protect and release the dexamethasone in a time delayed response. Therefore, we expected that drug effect would be greater.

We can summarize our results as such; mice exposed to Ova and pretreated with dexamethasone encapsulated in self-assembling nanoparticles had less lung inflammatory cells and lower cytokine levels than mice treated with equivalent doses of dexamethasone alone. Furthermore, the Dex-NP treated animals had lower lung resistance when challenged with methacholine and less structural airway changes. Collectively, the results show a better treatment response with the dexamethasone when it was contained within our unique self-assembling nanoparticles.

The inflammation of asthma is a Th2 lymphocyte mediated process. The Th2 cytokines IL-4 and IL-13 are critical to the initiation and potentiation of the airway inflammation and drivers of the remodeling response including mucous production, smooth muscle cell hypertrophy and epithelial cell proliferation. Both IL-4 and IL-13, and particularly in combination, are active targets for future therapies, though the IL-4 clinical trials were unpromising in the past [19]–[21]. In the Ova exposure mouse model, we would expect to see decreased production and secretion of these characteristic cytokines more so than non-specific inflammatory cytokines such as IL-6, TNF-α, and GM-CSF. Our lung lavage cytokine results are consistent with this notion as we found that IL-4 and IL-13 were significantly lower in the Ova Dex-NP treated mice compared to the Ova PBS control animals. There were no significant differences among the Ova treated groups in the discussed non-specific inflammatory cytokines. Interestingly, while IL-13 levels were lower in both Ova Dex and Ova Dex-NP treated groups compared to the Ova PBS controls, IL-4 was lower in the Ova Dex-NP animals only. IL-4 is a key cytokine in allergic inflammation and asthma. Not only does IL-4 stimulate isotype switching of B lymphocytes and IgE production, but it plays an important role in the recruitment of mast cells to the airway and endothelial vascular cell adhesion molecule-1 (VCAM-1) expression [22], which is critical to the migration of eosinophils and other leukocytes to the airway. IL-13, in turn, signals airway epithelial, goblet and smooth muscle cell changes that define the chronic remodeling response [17], [23], [24]. Our data is consistent with a pronounced suppression of Th2 lymphocyte mediated inflammation and airway remodeling.

It is intriguing to hypothesize that the nanoparticle-protected dexamethasone enabled a more potent Th2 lymphocyte inhibitory effect, perhaps through better lung deposition and the delayed release of drug. Furthermore, the potential to improve the nanoparticle formulation by adding a ligand that would increase the specificity towards lymphocytes, for example, would be appealing. The need to deliver drugs to target organs in a specific and efficient manner, at the lowest dose achievable, has allowed for the innovation of nanoparticles and nanocarriers of diverse properties. Lipid or hydrophobic nanocarriers have been developed which allow the delivery and deployment of hydrophobic drugs to target tissues, at lower doses and with higher efficacy. This would be possible with the addition of compound such as LLP2A, a high affinity α4β1 integrin antagonist that we have previously tested in this model [5], possibly for inhaled therapy.

In the field of cancer therapy, lipid nanocapsules appear as promising vehicles for the delivery of hydrophobic drugs such as docetaxel [25]. Docetaxel-loaded nanocapsules had earlier cancer anti-proliferative effects and apoptosis in MCF-7 breast cancer cells. Transfer of this technology to benign, chronic diseases like asthma has been slow, partly because of concerns with long term toxicities; however, there are some recent precedents with nanomicelles. Nanomicelles have been developed to carry various agents including beclomethasone dipropionate (BDP), as a carrier for delivery to the lungs. These phospholipid nanomicelles enhanced corticosteroid solubility by 1300 times its actual solubility, slowed down the release of drug, and demonstrated significant lung deposition [26]. The advantage of this system was the delivery of poorly soluble corticosteroids via nebulization while improving drug deposition. In another study, BDP in self-assembling pegylated phospholipid micelle for lung delivery showed excellent biocompatibility of both empty and drug-loaded systems when evaluated in human bronchial epithelial cells and erythrocytes [27]. In asthma, the potential to increase the potency of steroids in the lung in a safe and efficient manner, while lowering the total concentration delivered would be a significant step forward.

Concern regarding drug toxicity remains a major barrier to treatment and the expectation is that targeting encapsulated drug can minimize these toxicities. Nanoparticle delivery systems including micelles, liposomes, solid lipid nanoparticles, nanoemulsions, and nanosuspensions [28] are composed of physiological lipids and they are generally well-tolerated the human body. Despite this advantage, direct nanoparticle toxicity is a concern particularly for inhaled formulations which would be of interest in lung diseases like asthma. For example, the PEG^2k^-CA_4_ telodendrimer that we designed would never be administered as a blank, unloaded construct given the concerns with hemolysis, concerns that were previously identified in in vitro studies [6]. In our present asthma modeling studies, there is again enough correlative data with the LDH assay to raise this concern. LDH activity was significantly higher in the plasma of the empty nanoparticle (NP) treated animals compared to either the PBS control or Dex-NP treated animals. There were no significant differences in plasma bilirubin content among the groups, but this assay was complicated by the turbidity of the plasma in all NP and Dex-NP treated animals, which made the interpretation of the bilirubin readings difficult. Turbidity of the plasma was not an issue in the highly sensitive LDH assay. Overall, the results of increased plasma LDH in the empty NP treated animals is consistent with prior in vitro studies by our group and suggest that empty NP can cause hemolysis. This is not apparent in drug-loaded Dex-NP constructs. Taken together, drug loaded nanoparticles appear to ameliorate any potential for these carriers to trigger intravascular hemolysis. We believe that further safety and efficacy studies for consideration of a new Investigational New Drug application with this nanoparticle should continue but only with drug-loaded constructs. Hemolysis will be the safety concern that we will focus upon in future in vivo studies.

Outside of our findings, two important questions have been raised. Do these submicron particles penetrate the lung parenchyma and causes systemic injury, as is seen with asbestos and other particulate exposure? Two, does the body’s adaptive immune system generate an adverse response to nanoparticles over time? Recent studies investigating lung and systemic effects have used metal (titanium, zinc, silver) and silica containing nanoparticles [29], [30]. While systemic absorption is found with these heavy metal containing agents, the long term effects are unclear.

Perhaps a better comparator would be inhaled environmental particulate matter (PM) exposure in the 2.5–10 µm range; decades of studies have unraveled the time course for inflammatory cell recruitment and activation in the lung. Taken together, these studies show that acute particulate exposure leads to a transient loss of lung macrophages and adverse effects on macrophage function in the first 24 to 48 hours. For example, intra-tracheal instillation of ambient London PM10 into rats causes a decrease in macrophage numbers at 18 hours [31], a reduced macrophage migration response to zymogen-activated serum, and less phagocytic potential. Similarly, impaired alveolar macrophage chemotaxis has been observed after exposure to titanium oxide particles [32]. The reduction in macrophage numbers in lung lavage fluid appears to be maximal about six to twelve hours after exposure to PM_2.5–10_ in mice followed by a gradual recovery to baseline macrophage numbers by 18 to 24 hours [33]. The mechanism of acute toxicity of particulates on alveolar macrophage cells is not fully understood. Franzi and colleagues found a direct toxic effect of California wildfire PM on RAW264.7 cells, a murine macrophage cell line [34] that appeared to be mediated by oxidative damage. This notion is further supported by work by Chirino and colleagues who found a greater than 50% reduction in glutathione production in A549 lung epithelial cells exposed to PM10 from Mexico city [35]. Recovery from the initial adverse effects of particulate exposure on lung macrophage function requires the release and recruitment of bone-marrow derived macrophages. Taken together, we can surmise that upon exposure to environmental particulates, lung macrophages undergo a series of functional changes that critically impair their ability to clear particulates from the lung in the initial 24 to 48 hours. We can hypothesize that heavy metal based nanoparticles affect macrophages similarly, but further studies in this area are clearly warranted.

Several limitations to our study are apparent. First, we did not measure the pharmacokinetics of either the dexamethasone or the PEGylated nanoparticle compound in the mouse to determine optimal dosing. However, we believe that the magnitude of the anti-inflammatory effect of the compounds seen at the dosing interval chosen demonstrates efficacy. Second, we did not confirm the deposition of the synthesized compounds in the lung by in vivo imaging; however, we have shown previously that dye-labeled nanomicelles can be imaged in subcutaneous xenografts of tumors [36]. Third, the number of animals that were tested was small. We invested significant effort in assuring the purity of each nanoparticle protected drug and were constrained by the limited amount of these antagonists in each experiment. In this regard, these experiments should be considered early-stage development. Lastly, we did not design our study to be able to detect a potential anti-inflammatory effect of the empty nanoparticle alone. We were concerned about the potential for intravascular hemolysis and animal welfare. Given this, we performed only a single experiment of i.v. empty nanoparticles in a few animals. We cannot compare the independent effect of the empty nanoparticles and therefore cannot discount a contributing anti-inflammatory effect in mice exposed to ovalbumin.

## Conclusions

To summarize, we found that mice pretreated with dexamethasone encapsulated in self-assembling nanoparticles had less allergic lung inflammatory cells and lower inflammatory cytokine levels than mice treated with equivalent doses of dexamethasone alone. Furthermore, the Dex NP treated animals had better total lung resistance when challenged with methacholine and less of the structural airway changes that are seen with chronic airway remodeling. Collectively, the results show a better treatment response with the dexamethasone when it was contained in our unique self-assembling nanoparticles. However, it is important to note toxicity must be evaluated further if this work is to lead to clinical studies.
